# Modelling Associations between Public Understanding, Engagement and Forest Conditions in the Inland Northwest, USA

**DOI:** 10.1371/journal.pone.0117975

**Published:** 2015-02-11

**Authors:** Joel Hartter, Forrest R. Stevens, Lawrence C. Hamilton, Russell G. Congalton, Mark J. Ducey, Paul T. Oester

**Affiliations:** 1 Environmental Studies Program, University of Colorado, Boulder, Colorado, United States of America; 2 Carsey School of Public Policy, University of New Hampshire, Durham, New Hampshire, United States of America; 3 Department of Geography and Geosciences, University of Louisville, Louisville, Kentucky, United States of America; 4 Department of Sociology, University of New Hampshire, Durham, New Hampshire, United States of America; 5 Department of Natural Resources and the Environment, University of New Hampshire, Durham, New Hampshire, United States of America; 6 Oregon State University Extension Service, La Grande, Oregon, United States of America; Oregon State University, UNITED STATES

## Abstract

Opinions about public lands and the actions of private non-industrial forest owners in the western United States play important roles in forested landscape management as both public and private forests face increasing risks from large wildfires, pests and disease. This work presents the responses from two surveys, a random-sample telephone survey of more than 1500 residents and a mail survey targeting owners of parcels with 10 or more acres of forest. These surveys were conducted in three counties (Wallowa, Union, and Baker) in northeast Oregon, USA. We analyze these survey data using structural equation models in order to assess how individual characteristics and understanding of forest management issues affect perceptions about forest conditions and risks associated with declining forest health on public lands. We test whether forest understanding is informed by background, beliefs, and experiences, and whether as an intervening variable it is associated with views about forest conditions on publicly managed forests. Individual background characteristics such as age, gender and county of residence have significant direct or indirect effects on our measurement of understanding. Controlling for background factors, we found that forest owners with higher self-assessed understanding, and more education about forest management, tend to hold more pessimistic views about forest conditions. Based on our results we argue that self-assessed understanding, interest in learning, and willingness to engage in extension activities together have leverage to affect perceptions about the risks posed by declining forest conditions on public lands, influence land owner actions, and affect support for public policies. These results also have broader implications for management of forested landscapes on public and private lands amidst changing demographics in rural communities across the Inland Northwest where migration may significantly alter the composition of forest owner goals, understanding, and support for various management actions.

## Introduction

Wildfire affects 450,000 ha in the US. Wildfire protection funds for federal agencies have averaged $3.13 billion annually from 2002 to 2012, and suppression alone by federal agencies has averaged $712 million annually (1985–2013) [1–2]. The 2012 fire season was among the worst on record for many areas in the American West. Over a century of varied forest management strategies and practices have led to changes in forest structure, fire regimes, species assemblages, and riparian conditions, and in recent decades the Inland West in particular has seen unprecedented levels of tree disease and insects, mortality, and fire [3–5]. The number of occurrences of wildfire, their severity, and the cost to control them are on the rise as fires have grown bigger, fire seasons become longer, and damage more catastrophic [6–7]. The forecasted growth of wildfire in forests implies dramatic costs to neighboring communities that depend on forests [7–12]. At the same time, many of these communities that depend on forests and forest products for their cultural identity and as an economic engine have experienced dramatic changes due to the decline of active forest management. This includes not only timber harvest but also other treatments designed to meet objectives such as watershed protection and fuels reduction, particularly on public lands and parceled-out industrial forestlands [13]. Thus, it is important to understand whether people, particularly in resource-dependent rural communities, perceive risks associated with declining forest conditions and wildfire, and how those perceptions are associated with how people engage with the landscape.

Changing management objectives on federal lands in the 1990s de-emphasized wood fiber production and shifted toward goals of diversifying forest structure and habitat, creating fire-resilient landscapes, and restoring ecosystem functionality [3, 14–17]. While timber removal was necessary to meet these objectives, overall harvest volumes decreased by 70% in the 1990s and 2000s compared with previous decades [18, 20]. Such changes in forest management policy for federal lands, as well as changes in the milling infrastructure available to industrial and smaller private landowners, have led to changes in forest conditions. This loss of infrastructure affected the entire forestry production chain, including logging, trucking, and the service industries built to support timber-related activities across the West. Fire suppression has largely continued, and the marked decline in active management over the last 20 years, particularly on federal lands, has led to significant changes in forest conditions. Federal forests that were historically heavily harvested and actively managed are now becoming more dense and undisturbed, which contributes to increasing fuel loads, more standing and downed dead wood, and generally worsening conditions with regards to risks of fire, pests and disease [17, 21]. These changes in public lands have been exacerbated by long or ongoing droughts in many areas and changes in land ownership, land use, and land cover, which in turn increases the risk for large wildfire [22–23]. This is mirrored to a lesser extent on industrial and smaller private landowners’ forests [18–19]. The lack of forest products infrastructure has caused prices to decrease for raw timber products due to lower mill competition, and increased costs for commercial thinning and harvests because of increased hauling costs (N. Christoffersen, personal communication). Forest landowners are therefore now making decisions about their forests based on a more complicated mix of economic, social, and ecological factors.

The increasing number of wildfire ignitions and growing size of wildfires in recent years is a major concern for residents [24]. Such large scale disturbances expose vulnerabilities in communities and challenge relationships, trust, and confidence between different stakeholder groups [25]. Experience (direct or indirect) with wildfire heightens awareness and changes perceptions of risk and the possible outcomes of fire [26–27]. This is particularly true in communities neighboring federal lands, where the biggest wildfires occur [28–30]. Preventive action is critical to mitigating the effects of inevitable fire disturbances, and risk perception is a critical component to developing a response [31–33]. Perceived risk is constructed from experiences and social bases [34]. The ability to interpret perceptions and realities about wildfire risks and its mitigation it will be related to an individual’s knowledge and competencies [35] as well as their being engaged with common stakeholder groups [36]. Changing perceptions about wildfire can be attributed to interaction with landowners (e.g., building social networks, establishing relationships with newcomers, strengthening relationships with neighbors, discussing past experiences), engaging with knowledge providers (e.g., participation in extension or other learning activities), and by increasing landowner involvement in planning and facilitating preparedness in ways that increase capacity to prevent and respond to fire events [35–38]. In places where social capital through engagement is strong in their community, landowners are more likely to take action on their land, such as reducing fuel loads [28, 39]. Thus, knowing how to influence forest landowner action through changing both levels of understanding through education, experience, and engagement with other landowners is important for broader regimes of regional forestland management (i.e., for state and federal agencies, extension, non-profit organizations, and local, county and tribal governments) [27].

To better understand how landowner background influences perceptions about forest conditions and risk of wildfire we examined communities neighboring the Wallowa-Whitman National Forest in northeastern Oregon. In this study we examined the factors influencing the confidence of people in their own understanding of forest-related issues, and how levels of understanding relate to perceptions of forest conditions and wildfire risk. We hypothesized that the perceptions about general forest conditions, and forest health in particular, are related to an understanding of forest management. We expect that more self-professed knowledge regarding forest management will lead to a propensity to perceive risk of wildfire associated with unhealthy forests. We also examined the level of land owner engagement, as measured by their interest in actively managing and learning about their forests, as a factor separate from understanding that might affect perceptions. We expect that people who are more actively engaged a landowner is the more concerned they will be and more risk will be perceived with poor forest conditions. We also hypothesized that, given the demographic and landownership changes in this region [40] that perceptions may be different among residents, non-residents, and newcomers, which has implications for longer term forest management strategies for both private and public land owners.

### Study Area

**Fig 1:**
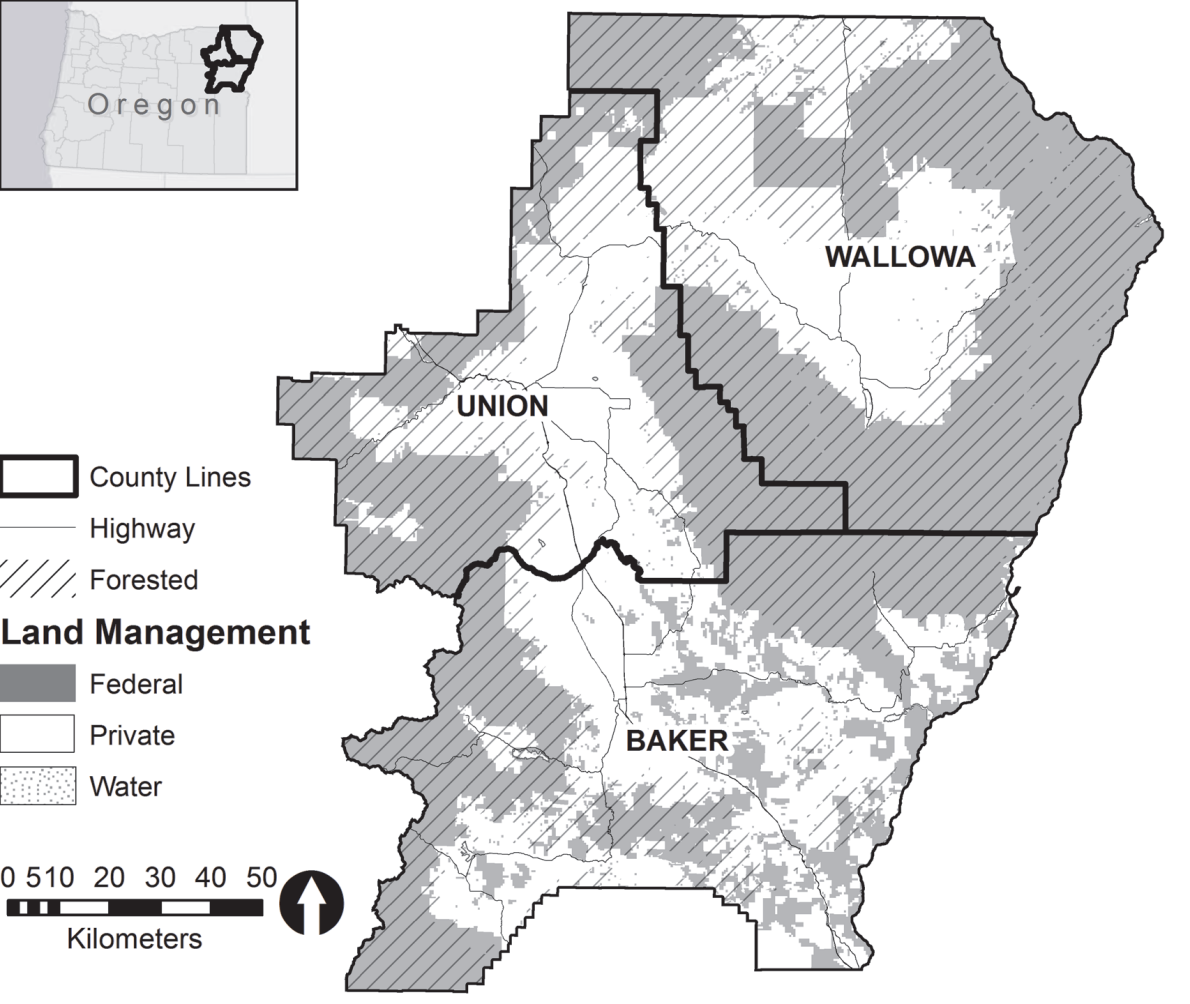
Wallowa, Union, and Baker counties sit in the most northeastern corner of the state of Oregon, USA. These three counties have a legacy of and currently are working landscapes, comprising agricultural, ranching, mining and commercial timber production, and a large percentage (53%) of federally managed land.

With a combined population of 48,489 [41], Wallowa, Union, and Baker counties are located in the remote, dry, and mountainous northeast corner of Oregon (Fig. 1). More than half the total land area (53%), including 70% of the forest lands, is managed by the federal government as the Wallowa-Whitman National Forest (which includes Hells Canyon National Recreation Area and Eagle Cap Wilderness Area) and other federal entities. A small number of species dominate these forests: predominately grand fir (*Abies grandis*), lodgepole pine (*Pinus contorta*), western larch (*Larix occidentalis*), and Douglas fir (*Pseudotsuga menziesii*) at the lower altitudes and wetter sites, ponderosa pine (*Pinus ponderosa*) on the drier and warmer sites, and subalpine fir (*Abies lasiocarpa*) and some Engelmann spruce (*Picea engelmannii*) and lodgepole pine at mid-altitude, wetter, and cooler sites. Timber production on public and private lands fell drastically over the past two decades, led by a decline of more than 90% (gross receipts) in federal-land harvests. Overall harvest decline since the 1980s, coupled with rising global competition, led to mill closures. In the state, milling infrastructure declined 74% (405 open mills in 1980 vs. 109 in 2010) [42]. Locally, all three industrial-scale facilities in Wallowa, five of eight in Union, and five in Baker have closed permanently, as well as mill closures in nearby Pendleton and John Day, OR and Walla Walla, WA [43]. Furthermore, the logging infrastructure (i.e., logging, trucking, and skilled labor) was reduced, and USDA Forest Service staffing was cut in half with several hundred jobs lost (along with their families and tax revenue due to out-migration) (N. Christofferen, personal communication, [44–45]).

At the same time, some retirees and others with remote careers or independent wealth have made new homes in this region, attracted by the area’s natural amenities and available land. Northeast Oregon exemplifies a broader trend in the West, where traditional livelihoods of farming and forestry are adjusting to newcomers, investors, and second homeowners. In Wallowa County, over 80% of the 100,000 acres of private non-industrial forests in the county, have changed hands since 1995 and have been purchased by non-residents [46].

In these places that depend so much on public lands for their identity and economic vitality, changes in forest policy and the rigidity of relevant laws and policies affecting forest management have resulted not only in lost jobs, but have altered forest dynamics. In northeastern Oregon, forests have been actively managed for over a century, but management on federal land struggles to move forward. The 100+ year legacy of fire suppression on the country’s national forests (which still succeeds in suppressing over 99% of all unwanted wildland fires during initial attack [47]) has contributed to unnaturally dense stands and understory vegetation with high fuel loads, leading to an increasing number of large, intense wildfires. The mix of fire suppression and dramatic, relatively quick reduction in active management in the 1990s has led to decreased heterogeneity, leaving the forest at greater risk for fire disturbance. Between 1980 and 2010 there were 115 recorded fires of 100 acres or larger within the Wallowa-Union-Baker area. These fires ranged in size from 100 to 79,149 acres in size, with a mean size of 6709 acres. The causes for these fires ranged from lightning to prescribed burns and other human-caused ignition sources. The average annual cost for suppression of these fires combined across the counties was $1,375,660 [48].

## Methods

### Surveys

Trained interviewers at the University of New Hampshire Survey Center conducted a total of 1,585 telephone interviews in September through early October of 2011. Land line phone numbers were selected at random within each of the three counties to obtain a fair cross-section of the public and interviews lasted about 10 to 15 minutes each. The overall response rate was 48%, calculated by the RR4 standard defined by the American Association for Public Opinion Research [49]. The survey deliberately oversampled forest landowners (n = 202), who might hold views different from those of the general public. Sampling weights in our analysis adjusted for the oversampling so they had no more than a proportional impact in our analyses. Forest landowners were defined as those owning ten or more acres of forest land. Researchers interviewed more than 500 residents in Baker and Union Counties each, and 365 in Wallowa County. Probability weights [50] compensate for the oversampling by county and forest ownership. Weights also allow minor adjustments based on comparison with census-estimated age, sex, and race tables for this region. Weighting also corrects for design bias related to household size and county population [34]. Of those that participated in the survey, only 68 respondents had permanent addresses outside Wallowa, Union, or Baker counties.

Between September and October 2012, we also conducted a mail survey of forest landowners in the same three counties to understand perceptions about forest management on both public and private land, threats to forests in the area, and actions they have taken to reduce those risks [43]. Landowners were identified using a forest classification of Landsat imagery aggregated to calculate forested area inside contemporary tax lot data from each of the three counties. The University of New Hampshire Survey Center mailed 2133 questionnaires to forest landowners (those who owned 10 acres or more forest land on a single parcel of their land), of which 454 (22%) were completed and returned. No follow-up of non-respondents occurred. In both cases, the University of New Hampshire Survey Center conducted the two separate surveys for this study. For the telephone survey, all consent was given over the phone by the respondent after the Survey Center representative read the informed consent statement. Since participation was anonymous, only verbal consent was given. For the mail survey, landowners were sent a survey along with a letter of informed consent. By completing and submitting their written responses, they consented to the study. The University of New Hampshire IRB (#4306) approved the two survey instruments and the mail and telephone informed consent protocols specifically for this study. The University of Colorado IRB (#14-0032) approved the use of these data for analysis.

### Analysis

**Fig 2:**
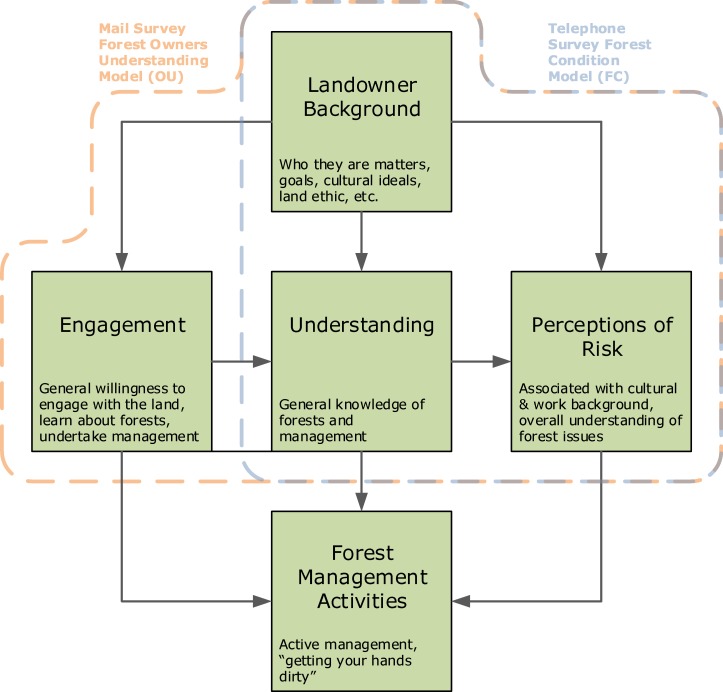
This diagram represents a general conceptual model relating activities on the landscape to land owners. The boxes represent various aspects of the landowner-landscape interaction, with the middle row representing knowledge, understanding, and opinions about the landscape, which in turn spur action or non-action with regards to active management activities on the landscape. Arrows represent hypothesized relationships. This general conceptual framework was used to craft survey questions for both the telephone and mail surveys and to specify two structural equation models testing some of the framework’s hypotheses.

To address the landowner-landscape interactions shown in our conceptual model (Fig. 2), we used the information gathered from both the general public telephone survey and forestland owner mail survey. For each survey we used structural equation modeling (SEM) to analyze direct and indirect relationships among individual background characteristics, measures of self-professed understanding, and perceptions about forest conditions and risks associated with wildfire on neighboring forested land. Path or structural equation models have been used to examine wildfire risk and engagement [27, 35] and these analyses showed that social processes and levels of understanding were significantly associated with a person’s perception of wildfire-related risk. Using such models gives us the ability to quantify the degree to which multiple interacting variables are associated with some latent or un-measured property indicated by one or more measured indicators [51]. However, a challenge associated with these methods is the need to correctly specify and evaluate candidate model structures that reflect potential underlying causal and correlative associations.

We used generalized path or structural equation modeling frameworks to analyze how landowner background affects understanding of forest management issues, which in turn affects perceptions about public lands (i.e., national forests) management. The telephone (general public) and mail (forest landowners) surveys used different questions. Consequently the models for these two datasets are conceptually parallel but divergent in their details. “Understanding,” the intervening variable, is quantified by a simple self-assessment item in the general-public survey. The forest-landowner survey employs a more elaborate measurement model for “understanding,” based on multiple actions. Perceptions about forest conditions and wildfire risk on public lands are quantified by a forest health question on the general public survey, and by a more specific item about fire danger on public lands on the forest-landowner survey. Thus, a separate model was built for each survey, related to components within the dashed lines (Fig. 2): Telephone Survey of the General Public, Forest Condition Model (FC model, blue box) and Mail Survey of forest Owners Understanding (OU model, orange box).

Path coefficients for both models were estimated using the R statistical modeling language [52], applying the *lavaan* R package [53]. For modeling dichotomous and ordinal endogenous variables, *lavaan* employs an ordered probit link function, with coefficients estimated by three-stage, diagonally weighted least squares, and mean/variance correction for calculations of test statistics (WLSMV) [54–55]. WLSMV is a robust alternative to maximum likelihood estimation for such models [55–56]. Survey items used as exogenous variables in theses SEM models were re-coded in some instances to simplify interpretation. Variables and coding for the FC model are shown in Table 1; those used for the OU model are shown in Table 2. The paths depicted in our conceptual model (Fig. 2) are evaluated empirically through observations, and in the case of the “Understanding” conceptual component in the OU formulation, a latent variable indicated by four survey items. These observations were regressed on surveyed information about the background, property ownership and experiences of survey respondents.

**Table 1:** Data summary for covariates of the “Forest Condition” (FC) model.

**Variable**	**Values**	**N**	**Raw %**	**Valid %**	**Cumulative %**
Gender	0: Male	634	40.00	40.00	40.00
	1: Female	951	60.00	60.00	100.00
	Missings	0	0.00		
Level of education					
	0: HS or less	445	28.08	28.47	28.47
	1: Tech/some college	499	31.48	31.93	60.40
	2: College grad	383	24.16	24.50	84.90
	3: Post-grad	236	14.89	15.10	100.00
	Missings	22	1.39		
Political party					
	0: Democrat	508	32.05	35.47	35.47
	1: Independent	201	12.68	14.04	49.51
	2: Republican	723	45.62	50.49	100.00
	Missings	153	9.65		
Lived here less than 10 years					
	0: Long-term	1223	77.16	77.65	77.65
	1: Newcomer	352	22.21	22.35	100.00
	Missings	10	0.63		
Wallowa county resident					
	0: Non-Wallowa resident	1220	76.97	76.97	76.97
	1: Wallowa resident	365	23.03	23.03	100.00
	Missings	0	0.00		
Own 10+ acres of forest land					
	0: No	1383	87.26	87.26	87.26
	1: Own 10+ acres	202	12.74	12.74	100.00
	Missings	0	0.00		
Forests same/more/less healthy than 20 years ago					
	0: Same/more	836	52.74	52.74	52.74
	1: Less	749	47.26	47.26	100.00
	Missings	0	0.00		
Understanding: forest health and management					
	0: DK/NA	59	3.72	3.72	3.72
	1: Little	311	19.62	19.62	23.34
	2: Moderate	790	49.84	49.84	73.19
	3: Great deal	425	26.81	26.81	100.00
	Missings	0	0.00		
Age					
	Min.	18.0			
	Max.	96.0			
	Median	61.0			
	Mean	60.3			
	Missings	24			

**Table 2:** Summary of used variables from the forest owner survey.

**Variable**	**Values**	**N**	**Raw %**	**Valid %**	**Cumulative %**
Gender	0: Male	307	67.47	74.15	74.15
	1: Female	107	23.52	25.85	100.00
	Missings	41	9.01		
Level of education					
	0: HS or less	69	15.16	16.59	16.59
	1: Tech/some college	95	20.88	22.84	39.42
	2: College grad	140	30.77	33.65	73.08
	3: Post-grad	112	24.62	26.92	100.00
	Missings	39	8.57		
Political party					
	0: Democrat	81	17.80	21.20	21.20
	1: Independent	107	23.52	28.01	49.21
	2: Republican	194	42.64	50.79	100.00
	Missings	73	16.04		
Ownership attained after 2000					
	0: Attained before 2001	279	61.32	71.72	71.72
	1: Attained after 2000	110	24.18	28.28	100.00
	Missings	66	14.51		
Wallowa county resident					
	0: Non-Wallowa resident	389	85.49	85.49	85.49
	1: Wallowa resident	66	14.51	14.51	100.00
	Missings	0	0.00		
What experience have you had with wildfire?					
	0: No wildfire	240	52.75	56.47	56.47
	1: Wildfire on own or neighbor's land	185	40.66	43.53	100.00
	Missings	30	6.59		
Do you or any close family members work professionally in the forest management or timber products industry?					
	0: No industry association	311	68.35	76.79	76.79
	1: Industry association	94	20.66	23.21	100.00
	Missings	50	10.99		
How much do you agree: Conserving natural resources means restricting their use and limiting access to them					
	0: Neither/mostly/strongly agree	113	24.84	27.83	27.83
	1: Strongly/mostly disagree	293	64.40	72.17	100.00
	Missings	49	10.77		
Understanding: forest health and management					
	0: DK/NA				
	1: Little				
	2: Moderate				
	3: Great deal				
	Missings				
Stand tending educational need (stand_ed)					
	0: Unmarked	162	35.60	35.60	35.60
	1: High need	39	8.57	8.57	44.18
	2: Medium need	93	20.44	20.44	64.62
	3: Low need	161	35.38	35.38	100.00
	Missings	0	0.00		
Forest pest/disease educational need (pest_ed)					
	0: Unmarked	199	43.74	43.74	43.74
	1: High need	73	16.04	16.04	59.78
	2: Medium need	93	20.44	20.44	80.22
	3: Low need	90	19.78	19.78	100.00
	Missings	0	0.00		
Pre-commercial thin educational need (thin_ed)					
	0: Unmarked	238	52.31	52.31	52.31
	1: High need	27	5.93	5.93	58.24
	2: Medium need	61	13.41	13.41	71.65
	3: Low need	129	28.35	28.35	100.00
	Missings	0	0.00		
Timber harvest and sale educational need (sale_ed)					
	0: Unmarked	233	51.21	51.21	51.21
	1: High need	36	7.91	7.91	59.12
	2: Medium need	52	11.43	11.43	70.55
	3: Low need	134	29.45	29.45	100.00
	Missings	0	0.00		
How high do you consider the risk of a dangerous fire on neighboring public land					
	0: Low/Don't Know	132	29.01	29.01	29.01
	1: Medium	112	24.62	24.62	53.63
	2: High	211	46.37	46.37	100.00
	Missings	0	0.00		
How often have you participated in OSU Forest Extension activities?					
	0: Never/Other	190	41.76	46.45	46.45
	1: 1/10 years	90	19.78	22.00	68.46
	2: 1/5 years	67	14.73	16.38	84.84
	3: 1/year	62	13.63	15.16	100.00
	Missings	46	10.11		
Full-time permanent residence in Wallowa, Union or Baker county					
	0: Non-resident	183	40.22	40.22	40.22
	1: WUB resident	272	59.78	59.78	100.00
	Missings	0	0.00		
How much do you agree: As a whole, public lands are managed well, thereby improving or maintaining forest conditions					
	0: Neither/mostly/strongly agree	166	36.48	40.89	40.89
	1: Strongly/mostly disagree	240	52.75	59.11	100.00
	Missings	49	10.77		
Age					
	Min.	28.0			
	Max.	92.0			
	Median	66.0			
	Mean	65.5			
	Missings	46			

### “Forest Conditions” (FC) Model

Both perception of wildfire risk and self-assessed understanding are important to engender support for forest management policies and activities in this region. We hypothesize that perceptions of declining forest conditions and wildfire risk increase with understanding or knowledge of forest conditions, even controlling for background factors (e.g., age, sex, income) of landowners within these rural, working landscapes. To test this hypothesis, we examine alternative “understanding” indicators from the forest landowner mail survey, viewing them as possible intervening or mediating variables that are related to respondent background and place characteristics, but also help to predict general views about forest management or health. The FC model applies these ideas.

In the telephone survey we asked respondents several questions of particular relevance to our conceptual model. These addressed the general features of our system including “Landowner Background,” “Understanding” of forest systems, and “Perceptions of Risk” (Fig. 2). First, “Do you think that the forests in your area are less healthy than they were 20 years ago, more healthy than 20 years ago, or is forest health about the same?” This question serves as an indicator for “wildfire risk perception” in the conceptual model since declining forest conditions increase risk of not only disease and pests but most visibly and dramatically wildfire in these arid, water-regulated coniferous forests. Since forests and the conditions of forests are so integral to the local livelihoods, culture and ecosystems of Wallowa, Union, and Baker (WUB) counties, the risk associated with worsening forest conditions have high salience. Worsening forest conditions (i.e., less healthy forests) in this context are generally associated with increasing stand density, over-stocking, and more downed, dead or dying trees which together relate to pest and disease outbreak, higher fuel loads and thus increased the likelihood of larger, more dangerous wildfires. A second indicator question from the telephone survey involves self-assessed understanding: “Regarding forest health and management, how much do you feel you understand about this issue—would you say a great deal, a moderate amount, only a little, or nothing at all?” This question is self-assessed rather than tested knowledge, so it represents a respondent’s self-confidence with an imperfect relationship to real comprehension.

### Forest “Owner Understanding” (OU) Model

We hypothesize that a landowner’s willingness to engage in forest-related issues and management is indicated by seeking information and knowledge. A landowner’s level of engagement in turn will likely affect perceptions about the conditions of nearby forests (see outlined relationships in Fig. 2). Since experience with forest land management is much less common among people who do not own forest land we did not pose direct questions related to active forest management on the general-public telephone survey. On the other hand, management has immediate relevance to forest landowners, and such questions were included on the mail survey.

**Fig 3:**
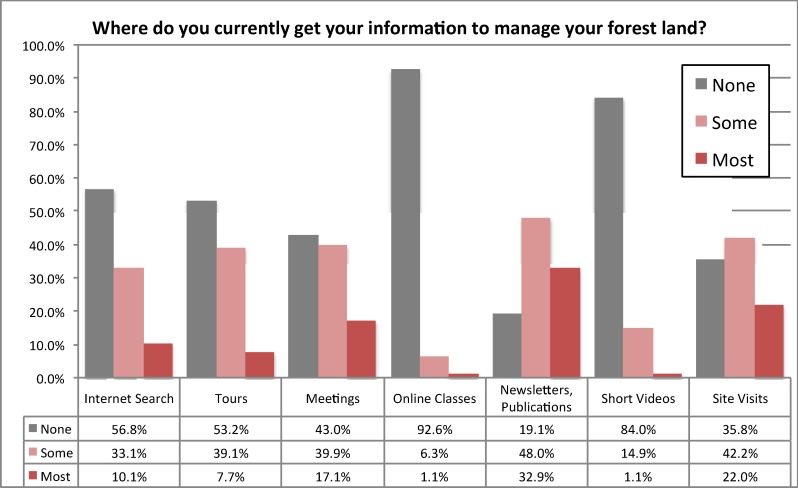
Mail survey question, “Where do you currently get your information to manage your forest land?

As a proxy for the “Engagement” component of our conceptual system (Fig. 2) we chose to use a mail survey item asking about how recently, if ever, the respondent has participated in Oregon State University extension activities. Extension provides a good proxy for engagement because these activities are the ones most relied upon for forest management information by respondents to the mail survey. Extension activities include workshops, tours, site visits, the OSU Master Woodland Manager program, one-on-one interactions with extension agents, online classes and training videos, and other public fora. Fig. 3 shows that much of the information people use comes from activities often, but not exclusively, organized by OSU extension: tours, meetings, newsletters, and site visits.

Similar to the FC model we fit a SEM with endogenous and exogenous variables. However, the key component of the OU model is the use of a more nuanced latent variable to represent forest understanding. This latent variable derives from mail survey questions regarding the educational need about four forest management practices: stand tending; pest and disease mitigation; pre-commercial thinning or fuel removal; and timber sales. These indicators were recoded as needed so that higher levels indicated lower need for education. With such coding they reflect increasing levels of forest understanding (Table 2). These indicators together were used to model the unobserved or latent variable of “Forest Understanding” which corresponds to the conceptual “Understanding” box of our system (Fig. 2).

While the conceptual model focuses specifically on perceived risk (Fig. 2) we conceived of this measure to include not only wildfire but more general risks associated with declining forest conditions. As such we wanted to align our analysis of the mail survey results with the analysis of the telephone survey FC model (which used a question about “forest conditions in your area”) and use a question regarding conditions as a proxy for perceived wildfire risk. To bolster that we did ask forest owners on the mail survey specifically how much of a risk they perceived regarding wildfire on neighboring lands of various owner groups (Table 2) and included that as a background predictor of perceptions about forest conditions. But it should be acknowledged that there are alternative ways to model perceived risk. We chose to incorporate forest owners’ broader view of forest conditions along with the implied potential risks to their community and livelihoods for the OU model using a question about public land management, “How much do you agree: As a whole, public lands are managed well, thereby improving or maintaining forest conditions.” We argue that this question is a good proxy for a scale of risk that extends beyond the conditions of neighboring lands and speaks to land owners’ general concerns about forest conditions due to the large percentage of publically owned forest in the region. It also minimizes the complexity of the model which is important due to the smaller number of mail surveys used in estimating the OU model. More complex formulations including measuring risk as a latent variable indicated by several forest condition-related questions were explored, however, the simpler model fit as well or better than the complex models and did not affect the associations of other non-risk-related covariates.

## Results

### “Forest Conditions” (FC) Model Results

**Fig 4:**
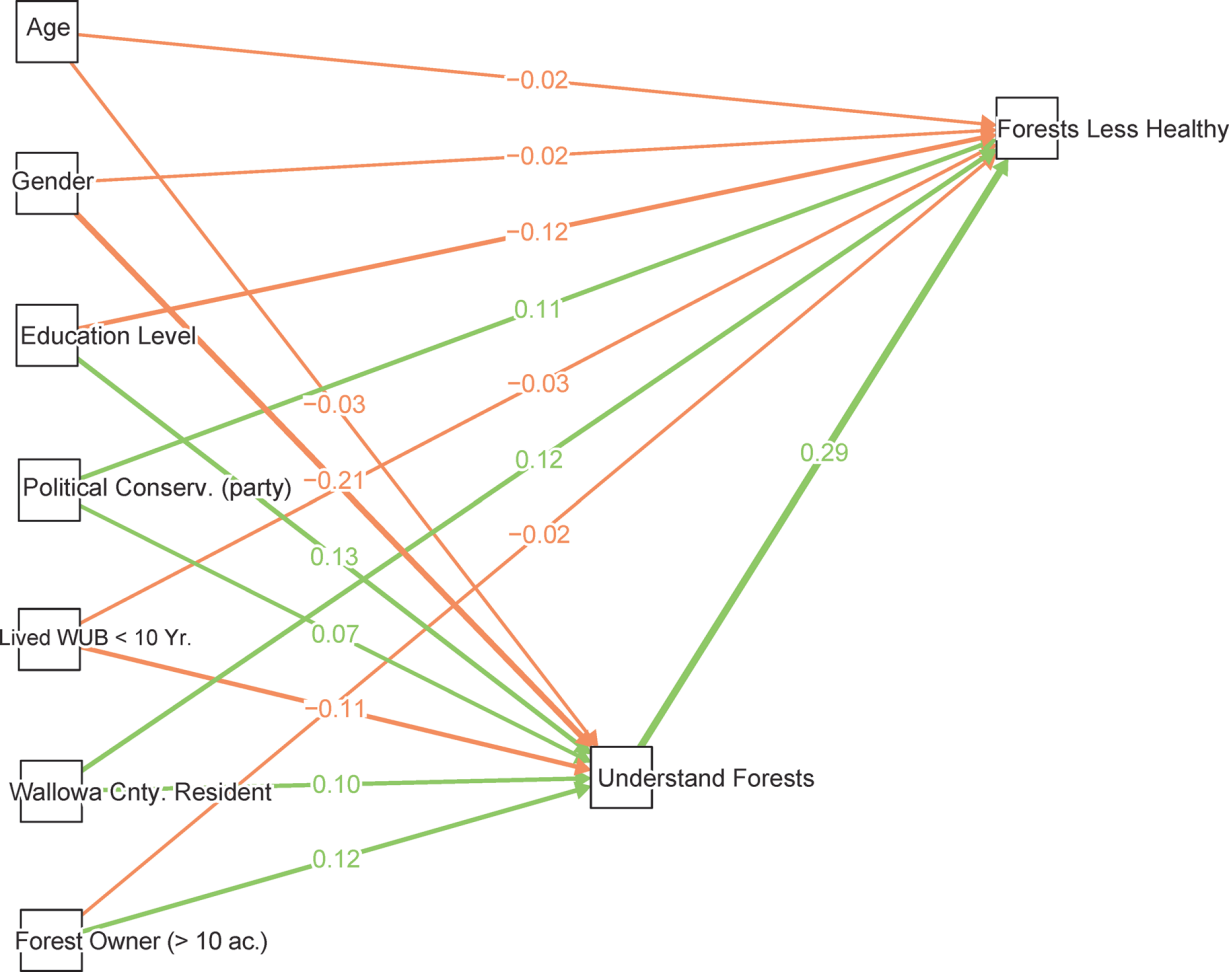
The path model that assesses relationships between forest understanding (*Understand Forests*), opinions about forest health (*Forests Less Healthy*) and various co-varying demographic indicators. These correspond to three boxes of the conceptual model highlighted as the Telephone Survey Forest Condition (FC) model. Landowner Background in Fig. 2 is represented by the demographic and landowner characteristics covariates on the far left, Understanding is represented by *Understand Forests* and Perceptions of Risk is represented by *Forests Less Healthy*. The arrows represent the modeled connections between exogenous predictors and endogenous variables of interest, *Understand Forests* and *Forests Less Healthy*, with an exception that forest understanding is also specified as a predictor for forest health. The arrows point towards variables being regressed on (dependent) and numbers represent the total standardized coefficient estimates with color representing the sign of the association. Since connections represent standardized values the relative strength of the association is indicated by the weight of the edge.

Table 1 summarizes survey results for the predictors and observed response variables in the forest condition model. Coefficients for this SEM are estimated from observed variables in the telephone survey data, including demographic and background factors to explain variability in forest understanding as well as opinions about forest health compared to 20 years ago. Findings are visualized as a path diagram with the estimated standardized coefficients indicating relative associations in (Fig. 3), and a plot of non-standardized coefficients with confidence intervals (Fig. 4). Statistical tables for this analysis can be found in the attached (S1 File).

We present the standardized coefficient estimates which approximate the observed correlation between variables due to presumed causal relations after all other correlations are accounted for that are included in the model structure [50]. Typically standardized coefficients are the result of converting both dependent and independent variables to z-scores, however where dependent variables are ordinal, like forest understanding, *lavaan* uses probit regression and standardization is only applied to predictors since the dependent latent variable upon which ordinal category thresholds are applied is already normalized. These standardized, estimated coefficients can generally be interpreted as the strength of the association relative to other covariates on the same units, and estimates how a one standard deviation unit change in a predictor will affect the underlying latent variable used as a threshold for ordinal categories. In contrast, the non-standardized coefficient estimates represent how a one unit change in the predictor of interest affects the probit transformed change in the predicted category and are generally more difficult to interpret.

The combination of endogenous and exogenous variables (e.g., the structural model), where no latent variables are estimated from exogenous indicators (e.g., a measurement model) is often termed a “path” model. Furthermore, this path model is a special case because the two endogenous variables, forest understanding and forest conditions compared to 20 years ago, are predicted by a nested subset of predictors, the only difference between the two being that *forests less healthy* is also regressed on *understand forests*. Therefore, the standard SEM “badness-of-fit” chi-square test statistic cannot be estimated because all exogenous variables are used for both *understand forests* and *forests less healthy*, and therefore is not reported (see S1 File. The relative fit vs. baseline model test statics like comparative fit index (CFI) and the Tucker-Lewis index (TLI) are also not informative (both were estimated to be 1.0, indicating a perfectly “valid” fit).

**Fig 5:**
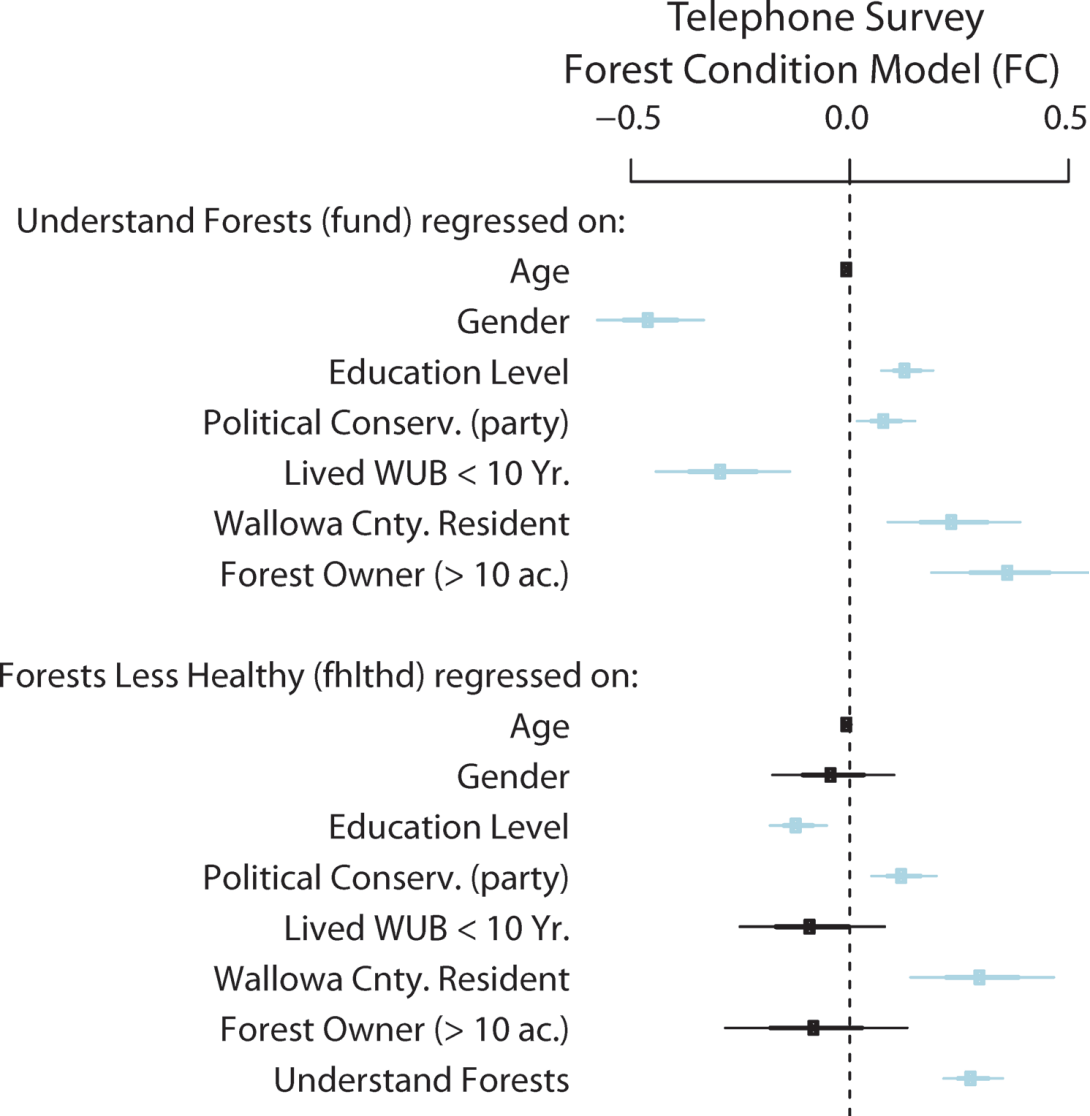
Non-standardized coefficients for the FC model are plotted with 95% confidence intervals indicated for each. Thick bars represent one standard error and thin bars represent two standard errors around the estimate. Confidence intervals that do not include zero are highlighted in blue.

By showing standardized coefficients (Fig. 4) for the fitted model we show how these covariates are related to both dependent variables of interest, and how the strongest predictor of opinions on forest health is self-professed forest understanding. This relationship in non-standardized terms is statistically significant and positive showing that higher levels of self-professed understanding of forests and forests management are associated with stronger opinions that forest health has declined over the last 20 years (Fig. 5). Another important result is newcomers (who have lived in the three counties less than 10 years) are less likely to perceive that forest health has declined. This effect occurs indirectly, however, via paths through forest understanding. In short, newcomer status (those that have lived in Wallowa, Union, or Baker counties less than 10 years) is negatively associated with understanding of forests, and understanding of forests positively associated with the perception that forests are less healthy now. So holding all other factors constant, we would expect a newcomer to perceive less risk from forest conditions on public lands

### Forest “Owner Understanding” (OU) Model Results

Results from the telephone survey and Forest Conditions model support the hypothesized relationship between understanding and opinions about forest conditions. But the telephone survey data contained no information about land owner engagement. We therefore wanted to explore the “Engagement” aspect of the conceptual relationship (Fig. 2) more explicitly. We quantified engagement using how often a land owner has participated in forestry extension activities (Table 2) as a proxy. We measured its conditional effect on forest understanding as indicated by stated educational needs for various forest management activities.

**Fig 6:**
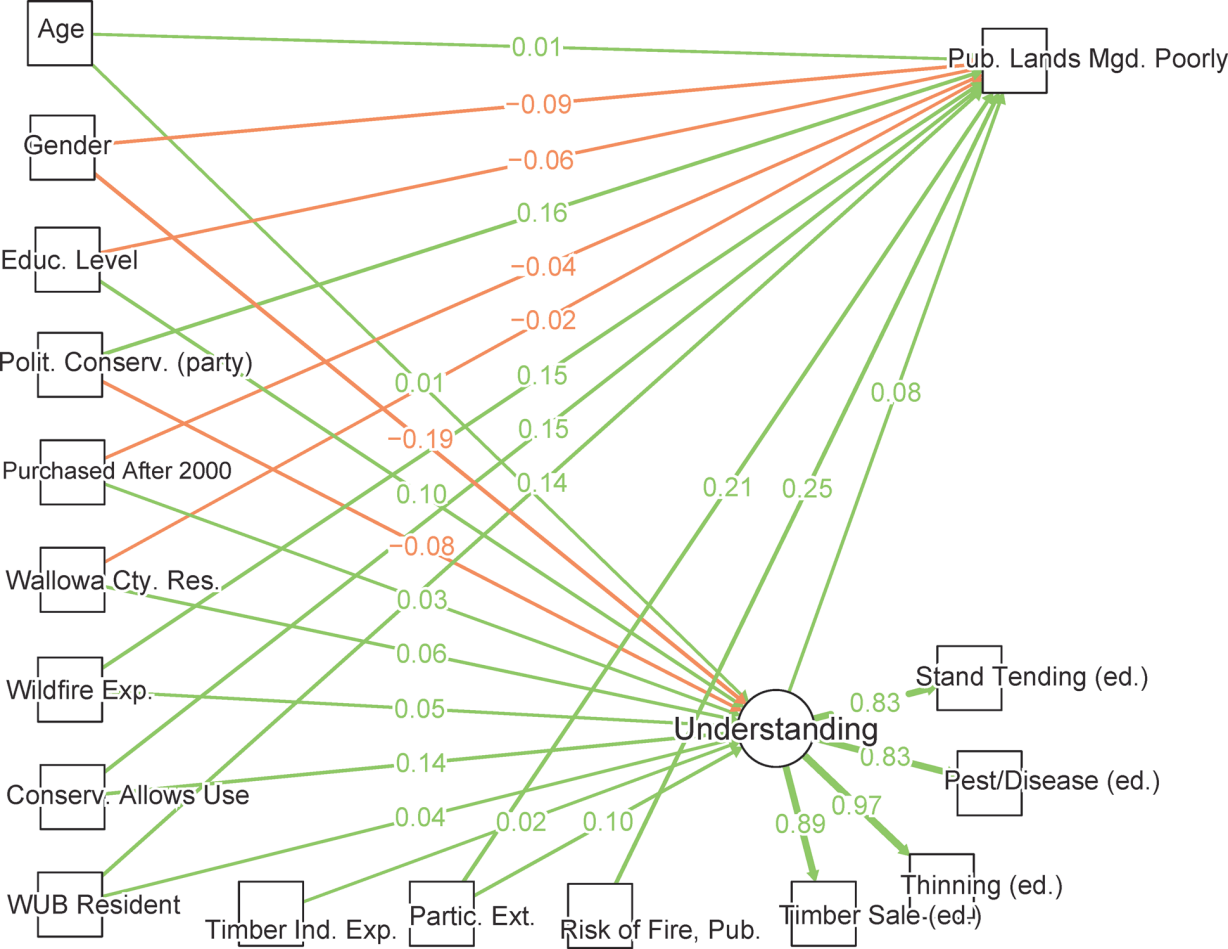
This is the structural equation model representing the conceptual components for the Forest Owner Understanding (OU) model outlined in Fig. 2. As in Fig. 3 the arrows represent connections between exogenous predictors and endogenous variables of interest, with arrows pointing towards the dependent variable, color representing the sign of the association, and relative strength (total standardized values) indicated by the weight of the edge and number for each connection. Landowner Background features from Fig. 2 are represented by the covariates along the left and bottom. We use participation in forestry extension activities in the last five years (*Partic*. *in Ext*.) as a proxy for Engagement. *Understanding* is represented as a latent variable indicated by questions asking about whether an owner states a need for education regarding certain forestry management activities, and Perceptions of Risk are represented by a question about whether, as a whole, public lands are managed poorly (Table 2) (*Pub*. *Lands Mgd*. *Poorly*). Unlike the FC model we specify a model including a latent factor of forest understanding, “Understanding”, which is measured by four indicator variables, *stand tending*, *pest/disease*, *thinning*, and *timber sale*.

**Fig 7:**
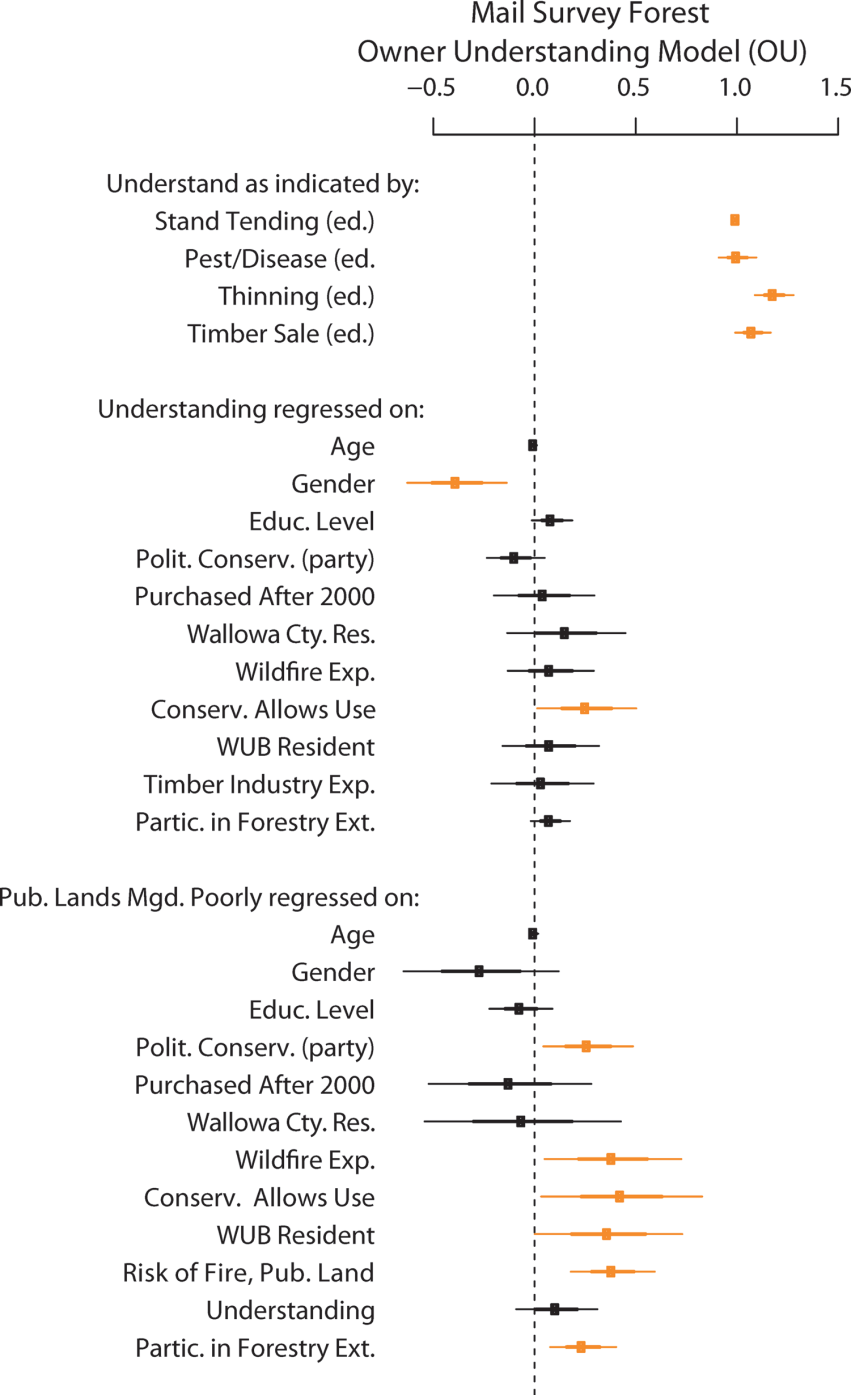
Non-standardized coefficients for the OU model are plotted with 95% confidence intervals indicated for each. Thick bars represent one standard error and thin bars represent two standard errors around the estimate. Recall that U*nderstanding* is a latent variable as measured by decreasing need for forestry extension education. It is regressed on demographic exogenous variables and included as a predictor for forest owners’ opinions on how well public lands are managed. Confidence intervals that do not include zero are highlighted in orange.

The results from this arrangement of exogenous and endogenous variables are presented as a path diagram (Fig. 6) and a plot of non-standardized coefficients with confidence intervals (Fig. 7). The overall, robust Chi-sq. “badness-of-fit” minimum function test statistic is 59.03 (p-value = 0.053, 43 d.f.), indicating that the level of fit is statistically better than nothing. We observe the robust estimated Comparative Fit Index (CFI) vs. the baseline null model is 0.994 and the Tucker-Lewis Index (TLI) is 0.990, both of which are relative fit indices where values near 1.0 are considered adequate. The root mean square error of approximation is near 0.034, which is well within thresholds for a “good” fitting SEM [56–57].

Model results show that the forest understanding latent factor is well described by the four educational indicators: stand tending, timber sale, thinning, pest and disease. Its relationships have criterion validity given how we expect stated educational needs to connect with knowledge about forest systems and their management. The strongest predictor among our exogenous variables is gender (Table 2, Fig. 6) because women are more likely to express greater educational needs (i.e. lower levels of current forest understanding). Also conditioned on all other covariates, forest landowners who strongly/mostly disagree with the opinion that, “conserving natural resources means restricting their use and limiting access to them” are more likely to express higher levels of forest understanding and higher levels of concern about publically managed forests.

Results show that the latent variable of understanding (standardized coefficient of 0.075) is not strongly associated with opinions about whether forest conditions have declined over the last 20 years (Figs. 6 and 7). Stronger associations were found for perceived risks of fire on neighboring public lands, standardized coefficient of 0.25); our proxy of engagement, frequency of participation in extension activities, (standardized coefficient of 0.21); and other background characteristics. Statistical tables for this analysis can be found in the attached (S2 File).

## Discussion

In northeast Oregon, public lands constitute a large percentage of landownership (53%) and a large percentage of the overall forested land in the study region (45%). It makes sense, therefore, that when thinking about conditions of all local forests, people generally think of public lands. Sentiments about forested lands and public lands in general are likely colored by changes in policy which massively decreased raw timber supply from public lands, subsequent loss of jobs in the forest products sector, and divestment of timber lands from the mills in the region. Thus, the perceptions about management of public lands should be a good barometer of the sentiment of the general public and forest landowners towards the majority of forests. Overall, there is strong concern about wildfire in northeastern Oregon, but it is particularly strong regarding public lands. Private industrial lands (~330,000 acres) and those held by small non-industrial forest owners are generally thought of by respondents to have much lower fire risk. Who these landowners are, their background and experiences, as well as participation in education (e.g., extension) on forest management all will shape their individual understanding of forests.

### Forest Conditions

For residents and land owners in Wallowa, Union and Baker Counties we found that forest conditions, management of forests and specifically the role they play on public lands are extremely important. Risk of wildfire is the highest ranked concern among the general public (73% in Baker County, 75% in Union, and 81% in Wallowa County) and for forest landowners as a group (79%) [58]. Generally, people perceive that forest conditions have worsened in the last 20 years. Despite most people claiming they knew a great deal about forests, there was some variability across the four levels measured for self-assessed forest understanding and based on prior research with these data we expected to see a relationship between understanding of forests and respondent’s opinions about how public lands are managed and their conditions relative to 20 years ago. We rationalized that a respondent’s opinion about whether forests were the same/more versus less healthy than they were 20 years ago would correlate strongly with a general perception of risk. This risk is a function of increased exposure to the hazard of wild fire and forest pests or disease due to declining forest health in nearby areas. Our results suggest that the conceptual model aligns with our chosen proxies for understanding and respondents’ perceptions about forest risks as do their associations.

We showed that self-professed understanding is associated with a variety of sources and background factors (gender, political affiliation) as well those who have a longer history in the area (those who purchased land before 2001), being a forest landowner and being a Wallowa County resident are all important predictors of greater self-assessed understanding taking all other tested factors into consideration. This self-professed understanding of forests is also an indicator of how well the general public may be engaged in forest-related issues.

As part of the conceptual model we hypothesized that respondents’ level of understanding of forest issues would color their perception of forest conditions, as well as their opinions about risks associated with fire, pests and disease. We also assumed these linkages would be conditioned on who our respondents were: demographically, culturally and based on their livelihood choices. We show that there is a strong, positive conditional association between forest understanding and perceptions that forest health has declined. This means that conditional on all of the other covariates, demographic and otherwise, the more people say they know about forests and forest management, the more likely they responded that forest health has declined over the last 20 years. Importantly, regardless of demographic background the more an individual claimed to understand about forests, the greater the likelihood that they thought forest conditions have decreased, which we assume increases the respondents’ perceived exposure to potentially dangerous wildfire and other hazards, thus equating to risk.

### Forest Owner Understanding

Paton and Tedim [35] suggest that the ability of community members to interpret forest fire risk and prepare to manage their risk will be a function of the degree to which they possess the competencies (i.e., through informal or formal education) that help them assess their own situation and act appropriately. People know where the knowledge gaps are and then seek ways of filling those gaps. Our mail survey was used to identify educational needs of forest landowners related to forest management activities—ladder fuel removal, pest and disease management, pre-commercial thinning, and timber sales—as a way of measuring the level of understanding of forest management. Thus, if a respondent reported a need for more education in a certain, there was a knowledge. Conversely if the landowner reported no educational need for those forest management activities, we assumed that there was a higher level of understanding of forest management. While many of the respondents participate in Oregon State University extension services in the last 10 years (54%), we found that general understanding was predicted by gender, views on conservation, and only weakly by education level and participation in extension activities.

The lack of a strong relationship between understanding and extension participation may indicate a more complex relationship between landowner background, educational needs and participation than the one explored in our research. There is a slight correlation, but this could be due to the way the latent factor of understanding was constructed or what it actually represents as indicated by expressed educational need. We assumed that landowners would choose to attend extension activities if they find their need for information strong, and not attend extension activities because they feel they have already gained that knowledge. In fact, people may be choosing to not attend because they truly have no need for this education, they are unaware of extension activities and the community it provides, or they are getting information related to forest understanding via pathways that our survey did not measure. When this mail survey is reapplied in further research, follow-up questions will be necessary to elicit the diverse reasons why education might not be needed.

We must note that these relationships of forest understanding with other covariates are not consistent with those that had a high degree of explanatory power for the FC model (e.g. level of education, political association, and whether the respondent lives in Wallowa County were significant predictors of understanding in the FC model, but not for OU). In the FC model (Fig. 4) we showed that those telephone survey respondents who owned 10 acres or more of forested land were significantly more likely to indicate a higher level of forest understanding, but conditional on that understanding there was no statistical association between forest owners and non-forest owners with regards to perceived forest conditions (i.e., wildfire risk). One working hypothesis is that a landowner’s willingness to engage on the landscape, signifying an interest which leads landowners to seek information and knowledge about land management, will change levels of understanding (Fig. 2). But a noteworthy result from the OU model is that we found little evidence of a conditional association between level of forest understanding and our indicator for overall perceived risk, public land managed poorly (Fig. 7).

This distinction should underscore two things. First, the mail survey was only of forest owners, which by itself was a strong predictor of self-professed understanding in the FC model. Second, while both ways of getting at understanding rely on it being self-professed, the latent factor used in the OU model is more highly focused on actionable understanding than the general question used in the FC model. Therefore, there may be a disconnect between the quality and nature of “understanding” that these two surveys are capturing. This is highlighted in the OU model output (Fig. 7) that understanding is not a good conditional predictor of peoples’ opinions of how public forest lands are managed. While political party, personal experience with wildfire, opinions about conservation, WUB county resident status, perceptions about risks of wildfire on neighboring public lands and participation in extension activities are all positively associated with a pessimistic view of public forest management (Fig. 7).

### Experience and Understanding Shape Perceptions

There is widespread interest in management of public lands in the Inland West because of its strong influence on working lands, tourism, demographic change, and cultural identity [59–60]. Working lands, and particularly in our case, working forests refer to forests managed for a combination of environmental and socioeconomic objectives [61–62]. Our results support that how local people perceive public lands as a whole is a good proxy for perceived risks that influence land owner decision-making. This overall perception is colored by who people are—their political affiliation, their experiences, and their background. Together these components form individual landowner identities. Lost jobs and mill infrastructure has strained reliance on traditional livelihoods of extraction in the area and forced some into bankruptcy, relocation, or finding alternative means to make a living. Political party, as in the telephone survey, is an important predictor, in agreement with much previous work in the area [34, 58]. Political party, in this region, seems to color local impression about how public lands are managed and the way that people think about federal lands. These demographic and background factors are one of the features that may shift throughout a population with in- and out-migration. Accompanying these social and demographic shifts over the last 30 years are diverse ecological changes. Forests in northeastern Oregon are characteristics of forests throughout the Intermountain American West, which are threatened by the risk of catastrophic insect outbreaks and wildfire [7, 9–11]. Certainly those with fire experience will have higher concern, which tends to influence behavior to mitigate that hazard [63]. Together the results from these two models show that we need a deeper understanding of how changing demographics and bases of experience in the Inland Northwest may affect perceptions about forest health. We find that who land owners are and how much they profess to understand vary significantly with perceived risks and opinions on forest conditions. As the population demographics and experiential bases change due to out-migration, general aging of rural populations, and amenity-based in-migration, among other drivers, we should expect large effects on the perceptions about forest conditions that can be disconnected from the actual forest conditions themselves. The opinions about forest conditions in other words are less likely driven by the actual conditions themselves, but on various factors, such as opinions about the conservation, levels of education and experience with wildfire, that are culturally and experientially motivated [64–65], which was also found in other a series of regional rural-US surveys [34, 66]. It is worth noting that our proxy for engagement, participation in extension activities is significantly associated with opinions about public land management, and the more recently a forest owner has participated in extension activities, conditioned on all other covariates, the more likely they are to think that public land management has worsened forest conditions in these three counties. This result in conjunction with the fact that extension participation is not highly associated with the “understanding” latent variable indicates that in future surveys a more thorough treatment of these conceptual areas is needed to test associations and causal relationships with a higher degree of certainty.

Initially, we hypothesized that interacting factors would have significant effects on the propensity to actually manage the land (Fig. 2). While our results largely support the relationships among “Landowner Background,” “Engagement,” “Understanding,” and “Perceptions of Risk” we do have mixed results especially among the relationship of “Understanding” and perceptions regarding forest conditions. Our results will inform future refinements of the design of the conceptual model (Fig. 2) and the choice of observed variables as indicators of these concepts.

Though small private land owners and their decisions about how to manage forests might affect relatively small areas of land on a per capita scale, collectively this represents a significant portion of the forested landscape in the Inland Northwest. Further, the accumulation of many small land-use decisions can have dramatic and long-lasting landscape-level impacts [67]. We are also left with the final question of how best can we affect land management outcomes, if as a scientific community we find that land management goals and actions are not adequately meeting the reality of forest condition changes? One option that has begun to be implemented is management across ownership and management jurisdictions for the entire working landscape [68]. Forest collaboratives, which are working partnerships between public and private organizations, and individuals who will work together on landscape management may offer a way forward for bridging logistical and cultural divides regarding forest management [69]. Collaboration has emerged as a popular means to address complex environmental problems (e.g., [36]), such as forest health in the western US [70]. Collaborative institutions create the opportunity for frequent and sustained interaction among landowners having diverse motivations and values [33] and in the face of a changing demographic of forest land owners increasing these opportunities may be critical.

## Conclusion

In forested landscapes of the West, dispersed settlement has been a growing trend. Many people are choosing to live out of town on small “ranchettes” [71–73], in rural places that are rich in natural amenities and offer opportunities for retirement or second home property development [74–75]. Migration also brought formerly urban populations into the rural northwest as landowners and seasonal or permanent residents [76]. At the same time, many private non-industrial (family-owned) forests have transferred from families who were financially dependent on timber harvests to retirees, amenity-seekers and others who value the forest more for its aesthetic and recreational properties than as a source of income [13]. Altogether, this ongoing ecological deterioration, declining commodity timber-production, changing management goals on public lands, the occurrence and prevalence of wildfire, changing demographics, and shifting local understanding of forest management have impacted the perceptions of wildfire risk.

We hypothesized that, given the demographic and landownership changes in this region [40], perceptions would be different among residents and non-residents. Our results supported this hypothesis among forest owners (WUB residents were more likely to have a pessimistic view of public land management). There is also a widespread perception among the general public and the forest landowners of the region that declining forest conditions and wildfire is a pervasive risk, especially wildfire which would occur on nearby national forest. We showed that among the general public, but not among forest owners alone, the more someone says they understand about forest management, after accounting for background factors like new ownership, education, and gender, the more pessimistic their views are on how forests in the area are doing. We also showed that newcomers are more likely to think positively about forest conditions and that forests on public lands in the area do not pose a high risk. This informs and supports our hypothesis that changing demographics including in-migration to the three counties may significantly alter perceived risks across communities as a whole.

This study also provides some insight on how land owner engagement, as measured by our proxy of participation in forestry extension activities, might relate to professed understanding and perceptions about forest health. While forest understanding as measured by desired extension education is not a strong predictor of opinions about public land management as a whole, participation in extension is strongly associated with more pessimistic views of public land management and we assume increased perceived risk. Increasing the penetration of forest extension services to new and changing demographics within these communities may be the leverage point with which forest conditions on private lands may further be improved by raising awareness of real declines in forest conditions. This could therefore mitigate real risks, not just those perceived on neighboring public lands, and furthering the potential for collaboration and shared decision-making for public-private partnerships for forest management in these forested landscapes. Forest collaboratives advocate for science-based restoration and management that considers broad ecological and public objectives. These collaboratives, much like extension, may have the opportunity and potentially the responsibility to influence these perceptions of forest conditions and wildfire risk by increasing engagement and understanding in order to change forest management activities and outcomes.

## Supporting Information

S1 File SEM model covariates and fit for the “Forest Condition” (FC) model. (DOCX)

S2 File SEM model covariates and fit for the forest “Owner Understanding” (OU) model. (DOCX)

S1 Dataset Clean and model-ready survey data used in “Forest Condition” (FC) model. (CSV)

S2 Dataset Clean and model-ready survey data used in “Owner Understanding” (OU) model. (CSV)
