# Supplementary material for: Modelling Associations between Public Understanding, Engagement and Forest Conditions in the Inland Northwest, USA
Source: PLoS One. 2015 Feb 11;10(2):e0117975. doi: 10.1371/journal.pone.0117975 (PMC4324782; doi:10.1371/journal.pone.0117975)
Supplement: S1 File — (DOCX) [file pone.0117975.s001.docx]

Supplement 1. SEM model covariates and fit for the “Forest Condition” (FC) model

Lookup table for survey items, names used in figures and text and supplementary code and data representations for those items.

| Survey Question (See Table 2 for Coding) | Recoded Figure/Text Name | Supplementary Code and Data Name |
| --- | --- | --- |
| Age | *Age* | age |
| Gender | *Gender* | gender |
| Level of education | *Education Level* | educ |
| Political party | *Political Conserv. (party)* | party |
| Lived here less than 10 years | *Lived WUB < 10 Yr.* | newcomer |
| Wallowa county resident | *Wallowa Cnty. Reesident* | wallowa |
| Own 10+ acres of forest land | *Forest Owner (> 10 ac.)* | ownfor |
| Forests same/more/less healthy than 20 years ago | *Forests Less Healthy* | fhlthd |
| Understanding: forest health and management | *Understand Forests* | fund |

> ## Model of mail survey items as analog to phone survey, with understanding

> ## or educational latent variable:

> understanding <- '

+ # Measurement model:

+

+ # Regressions:

+ fund ~ age + gender + educ + party + newcomer + wallowa + ownfor

+ fhlthd ~ age + gender + educ + party + newcomer + wallowa + ownfor + fund

+

+ # Residual correlations:

+ '

>

> set.seed(2002)

> understanding_sem <- sem(understanding, data=model_data, ordered=c("fund", "fhlthd"))

> summary(understanding_sem, standardized = TRUE, fit.measures=TRUE)

lavaan (0.5-15) converged normally after 62 iterations

Number of observations 1414

Estimator DWLS Robust

Minimum Function Test Statistic 0.000 0.000

Degrees of freedom 0 0

P-value (Chi-square) 0.000 0.000

Scaling correction factor NA

Shift parameter

for simple second-order correction (Mplus variant)

Model test baseline model:

Minimum Function Test Statistic 244.412 235.134

Degrees of freedom 15 15

P-value 0.000 0.000

User model versus baseline model:

Comparative Fit Index (CFI) 1.000 1.000

Tucker-Lewis Index (TLI) 1.000 1.000

Root Mean Square Error of Approximation:

RMSEA 0.000 0.000

90 Percent Confidence Interval 0.000 0.000 0.000 0.000

P-value RMSEA <= 0.05 1.000 1.000

Parameter estimates:

Information Expected

Standard Errors Robust.sem

Estimate Std.err Z-value P(>|z|) Std.lv Std.all

Regressions:

fund ~

age -0.002 0.002 -0.937 0.349 -0.002 -0.025

gender -0.455 0.061 -7.469 0.000 -0.455 -0.211

educ 0.131 0.030 4.435 0.000 0.131 0.129

party 0.083 0.033 2.500 0.012 0.083 0.072

newcomer -0.290 0.077 -3.778 0.000 -0.290 -0.113

wallowa 0.238 0.076 3.148 0.002 0.238 0.095

ownfor 0.366 0.090 4.083 0.000 0.366 0.116

fhlthd ~

age -0.001 0.002 -0.642 0.521 -0.001 -0.021

gender -0.038 0.070 -0.540 0.589 -0.038 -0.018

educ -0.117 0.033 -3.601 0.000 -0.117 -0.118

party 0.124 0.037 3.329 0.001 0.124 0.110

newcomer -0.086 0.083 -1.034 0.301 -0.086 -0.034

wallowa 0.303 0.082 3.693 0.000 0.303 0.124

ownfor -0.077 0.104 -0.737 0.461 -0.077 -0.025

fund 0.282 0.034 8.344 0.000 0.282 0.290

Thresholds:

fund|t1 -2.108 0.166 -12.677 0.000 -2.108 -1.993

fund|t2 -0.889 0.147 -6.042 0.000 -0.889 -0.840

fund|t3 0.572 0.148 3.873 0.000 0.572 0.541

fhlthd|t1 -0.037 0.179 -0.209 0.834 -0.037 -0.036

Variances:

fund 1.000 1.000 0.894

fhlthd 0.920 0.920 0.867
