# Supplementary material for: Modelling Associations between Public Understanding, Engagement and Forest Conditions in the Inland Northwest, USA
Source: PLoS One. 2015 Feb 11;10(2):e0117975. doi: 10.1371/journal.pone.0117975 (PMC4324782; doi:10.1371/journal.pone.0117975)
Supplement: S2 File — (DOCX) [file pone.0117975.s002.docx]

Supplementary Table 2. SEM model covariates and fit for the forest “Owner Understanding” (OU) model

Lookup table for survey items, names used in figures and text and supplementary code and data representations for those items.

| Survey Question (See Table 2 for Coding) | Recoded Figure/Text Name | Supplementary Code and Data Name |
| --- | --- | --- |
| Age | *Age* | age |
| Gender | *Gender* | gender |
| Level of education | *Educ. Level* | educ |
| Political party | *Polit. Conserv. (party)* | party |
| Ownership attained after 2000 | *Purchased After 2000* | newowner |
| Wallowa county resident | *Wallowa Cty. Res.* | wallowa |
| What experience have you had with wildfire? | *Wildfire Exp.* | wildfire |
| Do you or any close family members work professionally in the forest management or timber products industry? | *Timber Ind. Exp.* | industry |
| How much do you agree: Conserving natural resources means restricting their use and limiting access to them | *Conserv. Allows Use* | conserv |
| Understanding: forest health and management | *Understanding* | fund |
| Stand tending educational need | *Stand Tending (ed.)* | stand_ed |
| Forest pest/disease educational need | *Pest/Disease (ed.)* | pest_ed |
| Pre-commercial thin educational need | *Thinning (ed.)* | thin_ed |
| Timber harvest and sale educational need | *Timber Sale (ed.)* | sale_ed |
| How high do you consider the risk of a dangerous fire on neighboring public land | *Risk of Fire, Pub.* | riskpub |
| How often have you participated in OSU Forest Extension activities? | *Partic. Ext.* | partic |
| Full-time permanent residence in Wallowa, Union or Baker county | *WUB Resident* | wubres |
| How much do you agree: As a whole, public lands are managed well, thereby improving or maintaining forest conditions | *Pub. Lands Mgd. Poorly* | pubmanwhl |

> ## Model of mail survey items as analog to phone survey, with understanding

> ## or educational latent variable:

> understanding <- '

+ # Measurement model:

+ understand =~ stand_ed + pest_ed + thin_ed + sale_ed

+

+ # Regressions:

+ understand ~ age + gender + educ + party + newowner + wallowa + wildfire + conserv + wubres + industry + partic

+ pubmanwhl ~ age + gender + educ + party + newowner + wallowa + wildfire + conserv + wubres + riskpub + understand + partic

+

+ # Residual correlations:

+ '

>

> set.seed(2002)

> understanding_sem <- sem(understanding, data=model_data, ordered=c("stand_ed", "pest_ed", "thin_ed", "sale_ed", "pubmanwhl"))

> summary(understanding_sem, standardized = TRUE, fit.measures=TRUE)

lavaan (0.5-15) converged normally after 77 iterations

Number of observations 322

Estimator DWLS Robust

Minimum Function Test Statistic 31.324 59.031

Degrees of freedom 43 43

P-value (Chi-square) 0.907 0.053

Scaling correction factor 0.677

Shift parameter 12.750

for simple second-order correction (Mplus variant)

Model test baseline model:

Minimum Function Test Statistic 3912.300 2642.045

Degrees of freedom 70 70

P-value 0.000 0.000

User model versus baseline model:

Comparative Fit Index (CFI) 1.000 0.994

Tucker-Lewis Index (TLI) 1.005 0.990

Root Mean Square Error of Approximation:

RMSEA 0.000 0.034

90 Percent Confidence Interval 0.000 0.016 0.000 0.054

P-value RMSEA <= 0.05 1.000 0.899

Parameter estimates:

Information Expected

Standard Errors Robust.sem

Estimate Std.err Z-value P(>|z|) Std.lv Std.all

Latent variables:

understand =~

stand_ed 1.000 0.855 0.829

pest_ed 1.003 0.046 21.639 0.000 0.858 0.831

thin_ed 1.184 0.048 24.829 0.000 1.013 0.970

sale_ed 1.079 0.044 24.566 0.000 0.923 0.890

Regressions:

understand ~

age 0.001 0.005 0.149 0.882 0.001 0.010

gender -0.383 0.123 -3.115 0.002 -0.448 -0.192

educ 0.087 0.050 1.729 0.084 0.101 0.104

party -0.093 0.071 -1.298 0.194 -0.108 -0.084

newowner 0.048 0.124 0.385 0.700 0.056 0.025

wallowa 0.157 0.146 1.078 0.281 0.184 0.064

wildfire 0.080 0.106 0.755 0.450 0.094 0.047

conserv 0.258 0.122 2.106 0.035 0.301 0.135

wubres 0.080 0.119 0.672 0.502 0.093 0.045

industry 0.039 0.126 0.312 0.755 0.046 0.019

partic 0.079 0.048 1.627 0.104 0.092 0.101

pubmanwhl ~

age 0.001 0.009 0.073 0.942 0.001 0.005

gender -0.264 0.192 -1.377 0.168 -0.264 -0.091

educ -0.067 0.078 -0.862 0.389 -0.067 -0.055

party 0.265 0.111 2.394 0.017 0.265 0.165

newowner -0.121 0.201 -0.604 0.546 -0.121 -0.044

wallowa -0.058 0.242 -0.239 0.811 -0.058 -0.016

wildfire 0.388 0.169 2.296 0.022 0.388 0.154

conserv 0.430 0.199 2.165 0.030 0.430 0.154

wubres 0.366 0.182 2.006 0.045 0.366 0.140

riskpub 0.387 0.104 3.732 0.000 0.387 0.250

understand 0.110 0.100 1.098 0.272 0.094 0.075

partic 0.240 0.081 2.950 0.003 0.240 0.211

Intercepts:

understand 0.000 0.000 0.000

Thresholds:

stand_ed|t1 -0.421 0.548 -0.767 0.443 -0.421 -0.408

stand_ed|t2 -0.189 0.548 -0.345 0.730 -0.189 -0.183

stand_ed|t3 0.436 0.547 0.798 0.425 0.436 0.423

pest_ed|t1 0.318 0.559 0.569 0.569 0.318 0.308

pest_ed|t2 0.775 0.560 1.383 0.167 0.775 0.751

pest_ed|t3 1.476 0.568 2.598 0.009 1.476 1.430

thin_ed|t1 0.071 0.583 0.122 0.903 0.071 0.068

thin_ed|t2 0.218 0.583 0.374 0.708 0.218 0.209

thin_ed|t3 0.642 0.584 1.099 0.272 0.642 0.615

sale_ed|t1 0.607 0.583 1.042 0.297 0.607 0.586

sale_ed|t2 0.833 0.584 1.426 0.154 0.833 0.804

sale_ed|t3 1.217 0.587 2.072 0.038 1.217 1.174

pubmanwhl|t1 1.428 0.694 2.058 0.040 1.428 1.142

Variances:

stand_ed 0.333 0.333 0.313

pest_ed 0.329 0.329 0.309

thin_ed 0.064 0.064 0.059

sale_ed 0.223 0.223 0.208

pubmanwhl 0.992 0.992 0.635

understand 0.667 0.050 0.912 0.912
